# Interventions to Reduce Hospital Length of Stay in High-risk Populations: A Systematic Review

**DOI:** 10.1001/jamanetworkopen.2021.25846

**Published:** 2021-09-20

**Authors:** Shazia Mehmood Siddique, Kelley Tipton, Brian Leas, S. Ryan Greysen, Nikhil K. Mull, Meghan Lane-Fall, Kristina McShea, Amy Y. Tsou

**Affiliations:** 1 Division of Gastroenterology, University of Pennsylvania, Philadelphia; 2 Leonard Davis Institute of Health Economics, University of Pennsylvania, Philadelphia; 3 Center for Evidence-Based Practice, University of Pennsylvania Health System, Philadelphia; 4 ECRI Evidence-based Practice Center, Center for Clinical Evidence and Guidelines, Plymouth Meeting, Pennsylvania; 5 Division of General Internal Medicine, University of Pennsylvania, Philadelphia; 6 Department of Anesthesiology and Critical Care, University of Pennsylvania, Philadelphia; 7 Division of Neurology, Michael J. Crescenz Veterans Affairs Medical Center, Philadelphia, Pennsylvania

## Abstract

**Importance:**

Many strategies to reduce hospital length of stay (LOS) have been implemented, but few studies have evaluated hospital-led interventions focused on high-risk populations. The Agency for Healthcare Research and Quality (AHRQ) Learning Health System panel commissioned this study to further evaluate system-level interventions for LOS reduction.

**Objective:**

To identify and synthesize evidence regarding potential systems-level strategies to reduce LOS for patients at high risk for prolonged LOS.

**Evidence Review:**

Multiple databases, including MEDLINE and Embase, were searched for English-language systematic reviews from January 1, 2010, through September 30, 2020, with updated searches through January 19, 2021. The scope of the protocol was determined with input from AHRQ Key Informants. Systematic reviews were included if they reported on hospital-led interventions intended to decrease LOS for high-risk populations, defined as those with high-risk medical conditions or socioeconomically vulnerable populations (eg, patients with high levels of socioeconomic risk, who are medically uninsured or underinsured, with limited English proficiency, or who are hospitalized at a safety-net, tertiary, or quaternary care institution). Exclusion criteria included interventions that were conducted outside of the hospital setting, including community health programs. Data extraction was conducted independently, with extraction of strength of evidence (SOE) ratings provided by systematic reviews; if unavailable, SOE was assessed using the AHRQ Evidence-Based Practice Center methods guide.

**Findings:**

Our searches yielded 4432 potential studies. We included 19 systematic reviews reported in 20 articles. The reviews described 8 strategies for reducing LOS in high-risk populations: discharge planning, geriatric assessment, medication management, clinical pathways, interdisciplinary or multidisciplinary care, case management, hospitalist services, and telehealth. Interventions were most frequently designed for older patients, often those who were frail (9 studies), or patients with heart failure. There were notable evidence gaps, as there were no systematic reviews studying interventions for patients with socioeconomic risk. For patients with medically complex conditions, discharge planning, medication management, and interdisciplinary care teams were associated with inconsistent outcomes (LOS, readmissions, mortality) across populations. For patients with heart failure, clinical pathways and case management were associated with reduced length of stay (clinical pathways: mean difference reduction, 1.89 [95% CI, 1.33 to 2.44] days; case management: mean difference reduction, 1.28 [95% CI, 0.52 to 2.04] days).

**Conclusions and Relevance:**

This systematic review found inconsistent results across all high-risk populations on the effectiveness associated with interventions, such as discharge planning, that are often widely used by health systems. This systematic review highlights important evidence gaps, such as the lack of existing systematic reviews focused on patients with socioeconomic risk factors, and the need for further research.

## Key Points

**Question:**

Which hospital-led interventions are associated with reducing length of stay (LOS) for high-risk populations?

**Findings:**

In this systematic review including 19 systematic reviews, 8 strategies for reducing LOS in high-risk populations were identified: discharge planning, geriatric assessment, medication management, clinical pathways, interdisciplinary or multidisciplinary care, case management, hospitalist services, and telehealth. Interventions were most frequently designed for older patients or patients with heart failure and were often associated with inconsistent outcomes in LOS, readmissions, and mortality across populations.

**Meaning:**

This systematic review found that across all high-risk populations, there are inconsistent results on the effectiveness associated with interventions to reduce LOS, such as discharge planning, which are often widely used by health systems.

## Teaser

This systematic review identifies and analyzes evidence regarding potential systems-led strategies to reduce hospital length of stay for high-risk populations.

## Introduction

Hospital length of stay (LOS) is a quality metric health systems use as a proxy of efficient hospital management. Reduction in LOS improves bed turnover, allowing hospitals to match demand with capacity for elective and emergent admissions, intensive care unit (ICU) care, and interhospital transfers.^1,2^ When demand exceeds capacity, emergency department crowding, ICU strain, and ward strain occur, all of which are associated with worse outcomes, including mortality.^3,4,5,6,7,8,9^ Classifying patient hospital stays into diagnosis-related groups with fixed reimbursements further incentivizes hospitals to improve LOS to maintain operating margins.^10^ However, important potential trade-offs between LOS reduction and postdischarge adverse outcomes (eg, readmissions, mortality) exist. Furthermore, prolonged LOS may be associated with negative patient and staff experience, as well as increased inpatient complications (eg, hospital acquired infections, falls), many of which may be preventable.^11^ Therefore, many hospitals aim to implement systems-level approaches to provide optimal care and a safe discharge while avoiding prolonged hospital stays.

Many strategies to reduce hospital LOS have been developed, including some targeting different aspects of patient management, such as clinical care (eg, enhanced recovery programs and early mobility programs^12,13,14,15^), and others focusing on staffing models^16,17^ and logistics of care coordination.^18,19,20,21,22^ Although evidence is limited, some interventions, such as enhanced recovery after surgery programs, have focused on elective admissions and have been reported as consistently associated with improved LOS for planned, scheduled surgeries.^23^ In contrast to elective admissions, much less is known about the effectiveness of interventions in unplanned hospitalizations, especially among populations at-risk for poor outcomes. This includes patients who are medically complex, such as older adults, patients with heart failure, or patients with other chronic comorbidities who are at increased risk for prolonged LOS. Similarly, such interventions may be less generalizable to patients with socioeconomic risk factors more likely to be affected by health care disparities and at increased risk for adverse events related to hospitalization^24^ and unnecessary delays in discharge.^25,26,27^ Furthermore, many hospitals within Learning Health Systems, including safety-net hospitals, serve populations at disproportionately high risk for prolonged LOS and often struggle to maintain operating margins as a result. Long-term financial viability of these hospitals is crucial to ensuring access to care for underserved populations.^5^ Therefore, the Agency for Healthcare Research and Quality (AHRQ) Learning Health System Panel identified a need to identify broad system-level interventions to reduce LOS among patients with high risk of prolonged LOS.^28^ To address this need, we performed an overview of systematic reviews to identify interventions intended to reduce LOS for high-risk populations and identify evidence gaps. In this systematic review, we summarize existing evidence, with a specific focus on hospitalized older adults and patients with heart failure.

## Methods

This systematic review is based on a technical brief performed by the ECRI-Penn Medicine Evidence-based Practice Center for the AHRQ. The protocol and final report are available elsewhere.^29^ We interviewed 7 key informants with expertise in health systems, health care delivery processes, high-risk populations, care model transformation, and hospital quality and safety and incorporated input to refine scope and aims. This report follows the Preferred Reporting Items for Systematic Reviews and Meta-analyses (PRISMA) reporting guideline for systematic reviews.

### Data Sources and Search Strategy

Medical librarians searched MEDLINE, PubMed, Embase, CINAHL, the Cochrane Library, and gray literature sources, including from government agencies and relevant stakeholder organizations, from January 1, 2010, through September 30, 2020, with updated searches through January 19, 2021. Search terms are provided in eTable 1 in the Supplement. The full search strategy is available in the full report, published elsewhere.^29^

### Study Selection

Abstracts and full-text articles were screened in duplicate by a methodologist and a clinician using DistillerSR (Evidence Partners) using predetermined criteria (Table 1). Based on input from key informants, we focused on 2 populations at high-risk for prolonged LOS: patients with socioeconomic risk factors (eg, underinsured or uninsured) and patients who are medically complex (eg, with frailty or multimorbidity) (Table 1). Systematic reviews were included if they examined acute unplanned hospitalizations in the United States, evaluated hospital-initiated interventions (such as case management or multidisciplinary team models), aimed to reduce hospital LOS, reported on LOS as a primary outcome, and were published in English. We also required systematic reviews to provide explicit search criteria and inclusion and exclusion criteria and assess risk of bias (ROB) for included studies. We excluded systematic reviews focused on admission for nonemergent elective procedures, utilizing interventions not initiated within hospital settings (eg, community-based programs or ambulatory care visits after discharge), or including more than 50% of studies from outside of the United States.

**Table 1.** Populations, Interventions, Comparators, Outcomes, Timing, Settings, and Inclusion and Exclusion Criteria

Category	Criteria
Population	Include hospitalized children and adults (including pregnant women) with ≥1 of the following risk factors for prolonged LOS, harms, or adverse outcomes: High levels of socioeconomic risk (eg, housing instability, social isolation, social vulnerability, social mobility, lack of social network, lack of social support, limited access to health care services or social services, rural settings) Medically uninsured, underinsured Hospitalization at safety-net, tertiary, or quaternary care institution Limited English proficiency Patients who are medically complex, including those with comorbid psychiatric or behavioral health conditions, comorbid substance use disorder, frailty, multimorbidity (≥2 chronic health conditions), and high-volume chronic disease conditions with significant risk of exacerbation or complications (including chronic kidney disease, diabetes, congestive heart failure, and chronic obstructive pulmonary disease) Exclude patients undergoing nonemergent or elective procedures
Interventions	Include interventions that are Initiated within the hospital Designed (at least in part) to evaluate LOS Examples include but are not limited to clinical pathways, enhanced recovery programs, discharge planning, case management, and multidisciplinary teams Exclude interventions that are Initiated, managed, or implemented by entities wholly external to the hospital setting Are not intended or expected to reduce LOS Examples include but are not limited to ambulatory clinic follow-up visits, community-based support resources, regulatory policies, and third-party reimbursement programs
Comparators	Include: Usual care, any comparison, or other active intervention
Outcomes	Include: Primary: LOS LOS index Secondary: Readmission Patient harms, such as mortality, hospital-acquired conditions Patient experience or satisfaction Patient functional return Clinician or staff satisfaction Resource use, including patient flow and discharge disposition Exclude Only describe cost-related outcomes without reporting LOS Cost-related outcomes that do not quantify valuations of both comparisons or alternative interventions (including usual or standard care) and both of their associated outcomes
Timing	Include: All
Setting	Include Acute care hospitalizations in general or pediatric hospitals Reviews of studies conducted in the United States Exclude Reviews focused solely on intensive care unit stays, emergency departments, or observation units Specialty hospitals (eg, psychiatric, ophthalmologic, orthopedic, cancer, rehabilitation, long-term acute care) Reviews of studies conducted solely outside the US

Abbreviation: LOS, length of stay.

### Data Extraction and Quality Assessment

A standardized data extraction form was used to collect patient population, hospital and intervention characteristics, comparators, and outcomes assessed. Included systematic reviews were required to meet several thresholds for quality, including explicit search strategies, inclusion and exclusion criteria, and ROB assessment; therefore, independent quality assessment of included reviews was not performed. LOS was extracted as the primary outcome, with associated balancing measures as secondary outcomes, including readmission and mortality rates. For each outcome, when available, we extracted strength of evidence (SOE) ratings provided by systematic reviews. If not provided, we assessed SOE using AHRQ Evidence-based Practice Center guidance.^30^

### Data Synthesis

We narratively summarized evidence for heart failure and older populations and developed an evidence map to summarize the direction of association, volume, and quality of existing data for interventions across key outcomes (ie, LOS, readmission, and mortality). We also highlighted important knowledge gaps in the evidence base.

## Results

Of 4432 potentially relevant studies, we included 19 systematic reviews in 20 publications, 1 of which was identified in our gray literature search.^20,31,32,33,34,35,36,37,38,39,40,41,42,43,44,45,46,47,48,49^ The most common reason for exclusion was that the systematic review did not describe a hospital-led intervention. Study selection is shown in the Figure. Characteristics of included systematic reviews are shown in eTable 2 in the Supplement.

**Figure.**
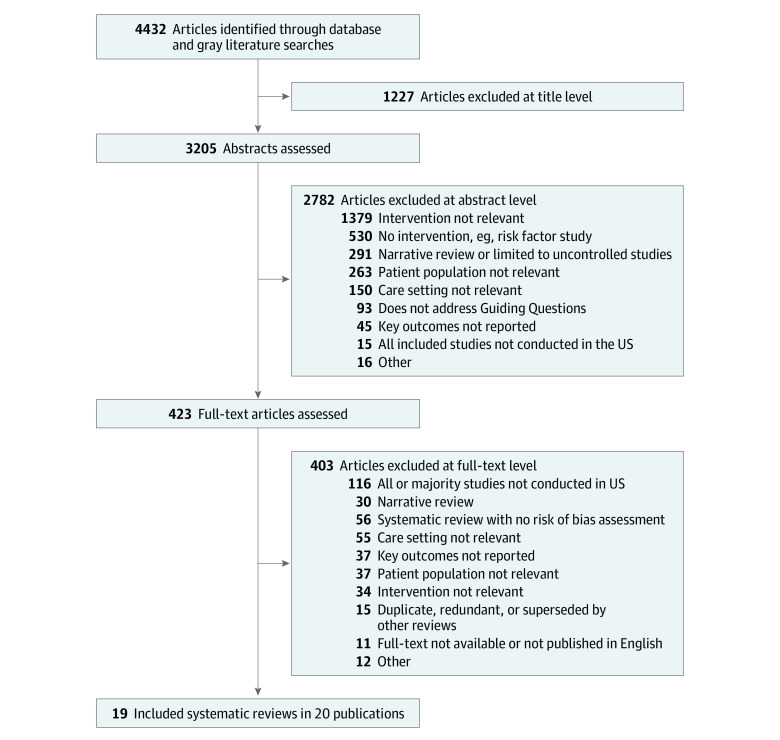
Diagram of Included Systematic Reviews

Of 19 systematic reviews analyzed, 10 included a mix of study designs (eg, randomized clinical trials [RCTs], observational cohort studies),^31,32,33,34,35,36,37,38,39,40,41^ 8 included only RCTs,^20,42,43,44,45,46,47,48^ and 1 included only retrospective cohort studies.^49^

Included systematic reviews addressed 8 types of systems-level hospital or health system interventions: discharge planning,^20,37,38,43,46^ geriatric assessment or consultation,^33,39,44,47,49^ medication management,^31,34,45^ clinical pathways,^36,42^ interdisciplinary or multidisciplinary care,^40,48^ case management,^35^ hospitalist services,^41^ and telehealth.^32^ There was an overlap of 10 individual studies across 8 systematic reviews evaluating discharge planning, geriatric assessment, or multidisciplinary care.^20,37,38,39,42,43,44,46,49^ The description and distribution of these categories is shown in Table 2, and individual study overlap is presented in eTable 2 in the Supplement.

**Table 2.** Description of Interventions to Reduce Length of Stay

Intervention	Included systematic reviews	Description of intervention
Geriatric assessment	Bakker et al,^33^ 2011	Multidisciplinary team with geriatrics consultation at various stages of patient care, including comanagement with surgical teams for preoperative optimization
	Patel et al,^39^ 2020	
	Ellis et al,^44^ 2017	
	Van Craen et al,^47^ 2010	
	Eagles et al,^49^ 2020	
Discharge planning	Zhu et al,^20^ 2015	Included an assessment of suitability for discharge, planning, implementation, and/or post-discharge follow-up. Follow-up care involved a phone call within 24 h of discharge, scheduling outpatient visits, home visits, and/or on-call services.
	Mabire et al,^37^ 2018	
	Mabire et al,^38^ 2016	
	Bryant-Lukosius et al,^43^ 2015	
	Goncalves-Bradley et al,^46^ 2010	
Medication management	Austin et al,^31^ 2020	Often targeted for high-risk medications, such as anticoagulants or antibiotics, with known adverse effects. Interventions included computerized entry systems, clinical decision support tools, dashboard utilization, pharmacist-led anticoagulation consultation services, and systematic education and feedback programs for patients.
	Gillaizeau et al,^34^ 2013	
	Frazer et al,^45^ 2019	
Clinical pathways	Kul et al,^36^ 2012	Included studies on multicomponent interventions, such as quality-improvement initiatives, including inpatient clinical pathway for heart failure management, standardized admission orders, education for staff and patients, or telephone surveillance after discharge
	Agarwal et al,^42^ 2019	
Multidisciplinary care	Pannick et al,^40^ 2015	Team coordination with inclusion of specialists during rounds, communication strategies, and task delegation for implementation of consultative recommendations
	Zhang et al,^48^ 2013	
Case management	Huntley et al,^35^ 2016	Directed by nurse case managers and included various strategies, such as medication review, family conferencing, education, home environment assessment, or referral to other services
Hospitalist service	White et al,^41^ 2011	Utilization of hospitalist physician staffing evaluated based on assessments of physician performance on quality of care
Telehealth	Baratloo et al,^32^ 2018	Hospital-based telehealth services for patients with stroke, linking hospital-based clinicians to outside clinical care teams

The clinical conditions reported included patients with heart failure and other patient groups, such as patients with acute stroke, pregnant women at high risk, and infants. Some systematic reviews synthesized data quantitatively for the LOS outcome, while others presented either a narrative synthesis or data from individual studies on reported LOS. Included reviews evaluated older adults and those with chronic conditions and quantitatively synthesized LOS and other outcomes (Table 2). Results from included systematic reviews are shown in eTable 1 in the Supplement, and those performing meta-analysis for LOS are presented in eTable 3 in the Supplement.

Systematic reviews reported limited information on the setting of included studies. There were 13 reviews that described interventions conducted in multiple types of hospitals, including academic medical centers, community hospitals, and, less frequently, Department of Veterans Affairs hospitals. Only 1 systematic review focused on trauma centers, and 6 reviews did not report hospital type. Only 5 reviews reported whether all included studies were conducted in urban, suburban, or rural settings: 3 included both urban and rural hospitals, 1 was limited to urban settings, and 1 included only rural hospitals. Few reviews reported hospital bed size or affiliation with a health system.

### Evidence Map

Table 3 summarizes quantitative findings from systematic reviews for the outcomes of LOS, readmissions, and mortality in an evidence map. The map provides an overview of direction of association, SOE, size and study designs contributing to the evidence base, and patient populations addressed. For most interventions, evidence for LOS reduction was inconsistent.

**Table 3.** Evidence Map of Systematic Reviews With Quantitative Synthesis^a^

Patient population	Intervention	Systematic review(s)	Study designs (No. of included patients)^b^	Outcome (strength of evidence)^c^		
				LOS	Readmissions	Mortality
Older adults	Discharge planning	Mabire et al,^37^ 2018	4 RCTs, 1 pre-post study, 1 cohort (2370)	↑ (L)	↓ (M)	NR
		Mabire et al,^38^ 2016				
		Goncalves-Bradley et al,^46^ 2016	12 RCTs (2193)	↓ (M)	↓ (M)	NR
		Bryant-Lukosius et al,^43^ 2015	3 RCTs (396)	↔ (L)	NR	↔ (L)
	Geriatric assessment	Eagles et al,^49^ 2020	2 retrospective cohort studies (5414)	↓ (M)	NR	↓ (M)
		Van Craen et al,^47^ 2010	7 RCTs (4759)	↔ (H)	↔ (M)	↔ (H)
		Ellis et al,^44^ 2017	11 RCTs (4346)	NR	NR	↔ (H)
Patients with heart failure	Discharge planning	Bryant-Lukosius et al,^43^ 2015	2 RCTs (495)	NR	↔ (L)	↔ (L)
	Clinical pathways	Kul et al,^36^ 2012	1 RCT and 4 observational studies (2095)	↓ (L)	↓ (M)	↓ (M)
	Case management	Huntley et al,^35^ 2016	8 RCTs and 1 observational study (1765)	↓ (M)	↓ (M)	NR
Patients with chronic conditions	Discharge planning	Zhu et al,^20^ 2015	5 RCTs (1912)	↔ (M)	↓ (M)	↓ (H)
	Medication management	Gillaizeau et al,^34^ 2013	8 RCTs and 1 observational study (n = 18 507)	↔ (L)	NR	NR
	Interdisciplinary care	Pannick et al,^40^ 2015	2 RCTs, 2 non-RCT cluster studies, 2 before/after studies (NR)	↔ (M)	↑ (L)	↔ (M)
Infants	Discharge planning	Bryant-Lukosius et al,^43^ 2015	2 RCTs (495)	NR	↔ (L)	NR
Pregnant women	Discharge planning	Bryant-Lukosius et al,^43^ 2015	2 RCTs (15)	↓ (M)	NR	NR
Patients with stroke	Telehealth	Baratloo et al,^32^ 2018	1 RCT, 2 prospective controlled studies, 6 retrospective controlled studies (2850)	↓ (M)	NR	↔ (M)

Abbreviations: L, low or very low; H, high; LOS, length of stay; M, moderate; NR, not reported; RCT, randomized clinical trial.

^a^ All reviews reported LOS data. NR in the LOS column indicates that the authors reported a narrative synthesis or results from individual trials instead of a quantitative synthesis. Narrative syntheses are not included here.

^b^ Number of patients included in quantitative synthesis for LOS. If LOS was not quantitatively synthesized, the number of patients for the outcome depicted with quantitative synthesis is reported.

^c^ Direction of association is indicated by arrows, with ↑ indicating increase; ↓, decrease; and ↔, inconclusive.

### Evidence Gaps

We found notable evidence gaps for high-risk populations. No systematic reviews focused on patients with socioeconomic risk factors (eg, housing instability, social isolation, no medical insurance or underinsuance, social mobility, lack of social network, lack of social support, limited access to health care services or social services, rural settings), limited English proficiency, or hospitalization at a safety-net or tertiary or quaternary care hospital. Furthermore, although systematic reviews did address certain populations of patients who were medically complex (including patients with chronic diseases, infants with high risk, and pregnant women with high risk), no systematic reviews assessed interventions for other key medically complex populations, such as patients with substance use disorder, comorbid psychiatric or behavioral health problems, or common chronic diseases, such as chronic kidney disease and chronic obstructive pulmonary disease.

### Older Patients

There were 9 systematic reviews (in 10 publications) that included older patients,^33,37,38,39,43,44,46,47,48,49^ with 4 systematic reviews focused on elderly patients who were frail. Of these, 5 systematic reviews performed quantitative synthesis of LOS and assessed discharge planning interventions (3 systematic reviews) and geriatric assessment or consultation (2 systematic reviews).

Discharge planning interventions (eg, early assessment of discharge suitability, establishing discharge plan and follow-up) had mixed results among older patients (Table 2). One systematic review by Mabire et al^37,38^ included 4 RCTs, 1 pre-post study, and 1 cohort study (including 2370 patients) assessing nursing-led discharge planning interventions, and found the intervention was associated with increased hospital LOS (weighted mean difference, 0.29 [95% CI, 0.24 to 0.35] days; low SOE). Although LOS increased, discharge planning was associated with improved readmission rates (odds ratio [OR], 0.57 [95% CI, 0.40 to 0.81]; *P* = .01), although a subanalysis specific to transitional care did not find an association with improvement (OR, 0.70 [95% CI, 0.38 to 1.27]). Another review by Goncalves-Bradley et al^46^ examined transitional care programs run by clinical nurse specialists across 12 RCTs (including 2193 patients) and found a decrease in LOS (mean difference, −0.73 [95% CI, −1.33 to −0.12] days; moderate SOE). A subgroup analysis focused on older surgical patients, including 2 RCTs (including 184 patients), found no change in LOS (mean difference, −0.06 [95% CI, −1.23 to 1.11] days). The third review by Bryant-Lukosius et al^43^ also evaluated a nurse-led intervention in 3 RCTs (including 396 patients) and found no difference in LOS (mean difference, −0.69 [95% CI, −1.95 to 0.56] days; low SOE).

There were 2 systematic reviews that quantitatively assessed outcomes associated with geriatric assessment or consultation interventions and also found mixed LOS outcomes. Eagles et al^49^ evaluated geriatric assessment performed by a geriatrician for reducing LOS for patients aged 65 years or older admitted to a trauma center. Based on 2 retrospective cohort studies (including 5414 patients), Eagles et al^49^ found that compared with standard care, geriatric assessment was associated with reduced LOS (mean difference, −1.11 [95% CI, −1.43 to −0.79] days; moderate SOE).^49^ Pre-post studies (including 7408 patients) from the systematic review by Eagles et al^49^ did not show improvement in mortality (unadjusted OR, 0.91 [95% CI, 0.70 to 1.18]), but cohort data comparing geriatric trauma consultation with standard of care (including 482 patients) did show improvement associated with consultation in inpatient mortality (unadjusted OR, 0.24 [95% CI, 0.12 to 0.52]; *I*^2^ = 0%; moderate SOE). In contrast, another review by Van Craen et al^47^ included 7 RCTs (including 4759 patients) and found no difference in LOS (mean reduction, 0.07 [95% CI, −0.11 to 0.26] days) or mortality at 12 months (relative risk [RR], 0.97 [95% CI, 0.88 to 1.08]; high SOE). Notably, the review by Van Craen et al^47^ focused on elderly patients who were frail, which may have represented a higher-risk population than other studies. Van Craen et al^47^ noted that the components and implementation of the intervention (geriatric evaluation and treatment unit) across included studies was heterogeneous.

### Heart Failure

We found 4 systematic reviews assessing 3 interventions (ie, discharge planning, clinical pathways, and case management) in patients with heart failure. However, only 2 of 4 systematic reviews meta-analyzed outcomes for LOS. One systematic review did not provide quantitative synthesis for LOS but found no difference in readmission (defined as unplanned readmission at 90 days) or mortality, although the intervention was associated with improved patient satisfaction.^43^

One systematic review performed a quantitative assessment of LOS for clinical pathway interventions. Kul et al^36^ included 1 RCT and 4 observational studies (including 2095 patients) and found that clinical pathway utilization was associated with reduced LOS (mean difference reduction, 1.89 [95% CI, 1.33 to 2.44] days; *I*^2^ = 42%; low SOE). There was also a reduction in readmission rate (RR, 0.81 [95% CI, 0.66 to 0.99]; *I*^2^ = 16%; moderate SOE) and inpatient mortality (RR, 0.45 [95% CI, 0.21 to 0.94]; *I*^2^ = 73%; low SOE).

A single systematic review by Huntley et al^35^ examined case management interventions. Huntley et al^35^ included 8 RCTs and 1 observational study (including 1765 patients) and found an association with decreased LOS (mean difference reduction, 1.28 [95% CI, 0.52 to 2.04] days; *I*^2^ = 63%; moderate SOE). Additionally, a sensitivity analysis excluding studies at high ROB found a mean reduction of 1.76 (95% CI, 1.23-2.29) days (*I*^2^ = 14%). Regarding readmission, Huntley et al^35^ found that case management interventions studied in 12 RCTs and 1 observational study (including 3346 patients) were associated with reduced readmission rates (RR, 0.74 [95% CI, 0.60 to 0.92]; moderate SOE) with increased heterogeneity (*I*^2^ = 69%), with similar results when high ROB studies were excluded (RR, 0.77 [95% CI, 0.61 to 0.96]; *I*^2^ = 68%).

A review by Bryant-Lukosius et al^43^ evaluated discharge planning interventions across multiple patient populations aged in their early to mid-70s, including patients with heart failure (2 RCTs with 495 patients). Although the systematic review did not perform quantitative synthesis of LOS for patients with heart failure, Bryant-Lukosius et al^43^ found no difference in readmission rates, defined as unplanned readmission at 90 days (RR, 0.81 [95% CI, 0.57 to 1.13]), and no difference in mortality (including 345 patients; RR, 0.76 [95% CI, 0.41 to 1.42]). However, the intervention was associated with improved patient satisfaction (including 403 patients; mean difference, 6.09 [95% CI, 3.55 to 8.63]; *P* < .001; moderate SOE).

## Discussion

This systematic review identified evidence for 8 hospital-based interventions targeting high-risk patient populations: discharge planning,^20,37,38,43,46^ geriatric assessment or consultation,^33,39,44,47,49^ medication management,^31,34,45^ clinical pathways,^36,42^ interdisciplinary or multidisciplinary care,^40,48^ case management,^35^ hospitalist services,^41^ and telehealth.^32^ As shown Table 3, aside from interventions for patients with heart failure, interventions were not consistently associated with reduced hospital LOS for medically complex populations. It is important to note that patients who are medically complex do not exist in silos, as older patients may also have chronic conditions, such as heart failure; therefore, identifying interventions that could reduce LOS across populations is important.

For patients with heart failure, clinical pathways and case management interventions were both associated with reduced LOS, readmission rates, and mortality.^36^ These findings are notable, since prior research has shown that interventions may improve LOS but worsen readmission rates or mortality.^50^ However, it is important to recognize the significant heterogeneity across studies in the systematic review by Kul et al^36^; similarly, another included narrative review on clinical pathways did not perform meta-analysis owing to increased heterogeneity.^42^ This suggests local context and resources (eg, team dynamics, hospital priorities, processes and staffing resources, and administrative support) are likely important factors in how successful interventions, such as clinical pathways or case management, are in streamlining care.^36,51,52^

Hospitalized older patients often have increased costs, complications, worse outcomes,^53,54^ and longer LOS compared with younger patients. For example, a study by Freeman et al^53^ estimated that compared with younger patients (aged 18-44 years), older adults (aged 65-84 years) had hospital stays that were a mean of 1.4 days longer. However, no intervention was consistently associated with reduced LOS for older patients. While 1 review found discharge planning was associated with a 0.73-day reduction in LOS in older patients,^46^ others found no association with reduced LOS, or even found an association with increased LOS.^37,38,43^ Inpatient geriatric assessment interventions also yielded inconsistent results. The largest review on this topic was unable to meta-analyze results owing to heterogeneity in study designs and settings across 11 RCTs.^44^ Studies’ settings included academic medical centers as well as community hospitals, and intervention components varied across studies. Thus, the variable associations with LOS across studies may reflect organizational differences, as LOS may vary depending on hospital factors, such as patient transfers, bed capacity, and demand. Future studies are needed to identify if interventions would provide more consistent benefits for particular types of hospitals or wards.

### Limitations

This systematic review has several important limitations. First, we used systematic reviews instead of primary studies to characterize interventions. Using systematic reviews facilitated searching for evidence across a broad range of populations and interventions and supported identification of evidence gaps. However, systematic reviews rarely reported key aspects of local environments (eg, demographic data on patient volume, bed size, payer mix, or other organizational capacity), which individual studies may have reported and would provide valuable context for health systems gauging feasibility and likelihood of success. Future systematic reviews assessing hospital LOS should certainly highlight these factors. Second, as systematic reviews inherently lag behind primary research, our evidence base may have missed interventions not yet captured in a published review. However, a targeted search of RCTs through January 19, 2021, identified no additional relevant studies pertinent to older or heart failure populations. Third, we did not formally assess systematic review quality; however, because systematic reviews were required to meet certain methodological standards for inclusion (ie, explicit search strategies, inclusion and exclusion criteria, and ROB assessment), all included systematic reviews met this threshold for quality.

## Conclusions

In this systematic review, we found 19 systematic reviews that identified 8 strategies for reducing LOS in high-risk populations: discharge planning, geriatric assessment; medication management; clinical pathways; inter- or multidisciplinary care; case management; hospitalist services; and telehealth. Identifying hospital-initiated interventions to reduce LOS without increasing readmissions or mortality is of interest to most hospitals and health systems. While many hospitals are searching for a one-size-fits-all solution to streamline care, our systematic review found that no single intervention was consistently associated with reduced LOS across all high-risk populations. A 2011 study by Hansen et al^55^ found that no single intervention was associated with reduced readmissions across broad populations. Since then, national initiatives have resulted in development of multifaceted approaches that include a range or combination of interventions that can be adapted for local context.^56^ Our findings suggest that efforts to reduce LOS, particularly for high-risk populations, could benefit from a similar approach. Future research assessing interventions for LOS reduction in high-risk populations or subpopulations should also consider implementation science measures to inform local adaption.

## Notes

Supplement. **eTable 1.** Embase and MEDLINE Search Terms in Embase Syntax **eTable 2.** Characteristics of Systematic Reviews on Reducing Length of Hospital Stay **eTable 3.** Summary of Findings for Length of Stay Meta-analyses
