# Supplementary material for: Interventions to Reduce Hospital Length of Stay in High-risk Populations: A Systematic Review
Source: JAMA Netw Open. 2021 Sep 20;4(9):e2125846. doi: 10.1001/jamanetworkopen.2021.25846 (PMC8453321; doi:10.1001/jamanetworkopen.2021.25846)
Supplement: Supplement. — eTable 1. Embase and MEDLINE Search Terms in Embase Syntax eTable 2. Characteristics of Systematic Reviews on Reducing Length of Hospital Stay eTable 3. Summary of Findings for Length of Stay Meta-analyses [file jamanetwopen-e2125846-s001.pdf]

Supplemental Online Content

Siddique SM, Tipton K, Leas B, et al. Interventions to reduce hospital length of stay in high-risk populations: a systematic review. *JAMA Netw Open*. 2021;4(9):e2125846. doi:10.1001/jamanetworkopen.2021.25846

eTable 1. Embase and MEDLINE Search Terms in Embase Syntax

eTable 2. Characteristics of Systematic Reviews on Reducing Length of Hospital Stay

eTable 3. Summary of Findings for Length of Stay Meta-analysese

This supplemental material has been provided by the authors to give readers additional information about their work.

© 2021 Siddique SM et al. *JAMA Network Open.*

eTable 1. Embase and MEDLINE Search Terms in Embase Syntax

Set	Concept	Strategy
1	Length of Stay	'length of stay'/exp OR ('hospital discharge'/exp AND 'time factor'/exp) OR 'los':ti,ab OR (((length OR duration) NEXT/3 stay):ti,ab) OR 'bed days':ti,ab OR (((length OR duration OR days) NEAR/3 hospital*):ti,ab) OR (((inpatient OR patient OR short) NEAR/1 (stay* OR throughput OR flow* OR days)):ti,ab) OR (((discharge* OR stay) NEAR/4 (delay* OR timely OR timeliness OR fast OR faster OR sooner OR quick* OR haste* OR rapid* OR early OR earlier OR reduc* OR decrease OR lessen OR speed*)):ti,ab) OR ((fast NEXT/1 track):ti,ab)
2	Population: Frail elderly	'frail elderly'/exp OR 'aged hospital patient'/exp OR 'very elderly'/exp OR 'dementia'/exp OR frail:ti,ab OR frailty:ti,ab OR dementia*:ti,ab OR alzheimer*:ti,ab OR 'hospital elder life program' OR 'older people assessment liason' OR opal
3	Population: Patients experiencing heart failure	'heart failure'/exp OR ((heart OR cardio* OR cardiac OR cardiogen* OR coronary) NEAR/3 (failure OR shock OR death OR infarct* OR arrest*)):ti,ab)
4	Combine sets	1 AND (2 OR 3)
5	Limit to English language and human studies	4 AND [humans]/lim AND [english]/lim
6	Exclude unwanted publication types	5 NOT (abstract:nc OR annual:nc OR book/de OR 'case report'/de OR 'case study'/de OR conference:nc OR 'conference abstract':it OR 'conference paper'/de OR 'conference paper':it OR 'conference proceeding':pt OR 'conference review':it OR congress:nc OR editorial/de OR editorial:it OR erratum/de OR letter:it OR note/de OR note:it OR meeting:nc OR sessions:nc OR 'short survey'/de OR symposium:nc)
7	Limit to Systematic Reviews	6 AND ('systematic review'/de OR 'meta analysis'/de OR (systematic* NEAR/2 review*) OR metaanalysis OR metaanalyses OR (meta NEXT/1 (analysis OR analyses)) OR Cochrane)
8	Limit to items published between 2010 and January 19, 2021	7 AND [01-01-2010]/sd NOT [19-01-2021]

*We updated the prior English-language literature searches conducted through 30 September 2020 by searching MEDLINE and EMBASE (via EMBASE.com), In-Process MEDLINE and PubMed-unique content (via PubMed.gov), and CINAHL for specific patient populations through 19 January 2021. See report for full search strategy (https://effectivehealthcare.ahrq.gov/products/hospital-length-of-stay)

eTable 2. Characteristics of Systematic Reviews on Reducing Length of Hospital Stay

**Reference**	**Objective**	**Search Timeframe**	**Inclusion/Exclusion Criteria**	**Primary Studies: N, Design**	**Age, No. of studies**	**Primary dx & comorbidity, No. of studies**	**GRADE or Similar Analysis**
**Austin et al. 2020 (30)**	Examine which electronic medical record interventions have improved safety and quality of therapeutic anticoagulation in an inpatient hospital setting	Inception to September 2018	Included: Studies published in English with pediatric and adult inpatients; EMR compared to routine care; reporting at least one outcome of interest	27 total studies: 3 RCTs, 4 cohort studies, 20 pre/post observational studies (N=not reported)	>18 years	Types of anticoagulants assessed: Unfractionated heparin: k=9 Vitamin K antagonists: k=8 Combination of anticoagulants: k=8 studies Low molecular weight heparins: k=2	No
**Agarwal et al. 2018 (41)** *Note: Laramee 2003 and Rich 1995a also included in Goncalves-Bradley 2016 and/or Bryant-Lukosius 2015	Examine the effect and quality of evidence for hospital-based HF quality improvement interventions on process of care measures and clinical outcomes among patients with acute HF	Inception to February 6, 2017	Included: RCTs or quasi-randomized trials of HF quality improvement interventions testing effect of individual or combined interventions (e.g., audit and feedback reporting systems, admission and discharge checklists, chart case management, patient educational or behavioral change materials, healthcare quality training that was directed at the hospital system,	14 RCTs (N=96,913) *(N=75,664 for 6 US RCTs) reporting outcomes of interest	67.5 to 79.3 years	Heart failure	Yes, GRADE (For the most part, outcomes from specific RCTs of interest for this report were not graded separately)

© 2021 Siddique SM et al. *JAMA Network Open.*

Reference	Objective	Search Timeframe	Inclusion/Exclusion Criteria	Primary Studies: N, Design	Age, No. of studies	Primary dx & comorbidity, No. of studies	GRADE or Similar Analysis
			doctors, nurses or allied health professionals or information management systems				
**Baratloo et al. 2018 (31)**	Examine effects of telemedicine on treatment times and clinical outcomes of acute stroke care	May 2017	Included: Original prospective and retrospective studies, individuals with AIS, telestroke-based systems, bedside (face to face) care as a comparator, studies investigating outcomes of interest<br><br>Excluded: Single-arm studies, studies reporting irrelevant outcomes, conference abstracts	26 total studies:<br><br>2 RCTs,<br><br>8 prospective observational studies,<br><br>16 retrospective observational studies (N=6,605)	Mean range: 60.1 to 80 years	Tissue plasminogen activator treated patients with acute ischemic stroke	No
**Bryant-Lukosius et al. 2015 (42)**<br><br>*Note: Kennedy 1987, Laramee 2003, Naylor 1994, 1999, and 2004 also included in Goncalves-Bradley 2016, Zhu 2015, Mabire 2017, and/or	Examine the clinical effectiveness and cost-effectiveness of clinical nurse specialists transitional care	1980 to<br><br>July 31, 2013	Included: Published and unpublished RCTs comparing CNS-led transitional care to usual care. Intervention was delivered by a master's prepared CNS<br><br>Excluded: Studies of outcomes that could not be solely attributed to the CNS; the control group was exposed to a CNS; or did not include a measure of	13 RCTS (N=2,463)	Patients with heart failure: mean range: 70.7 to 76 years<br><br>Elderly hospitalized patients: mean range: 74.4 to 80.3 years<br><br>High-risk pregnant women and	Patients with heart failure: k=3<br><br>Elderly hospitalized patients: k=5<br><br>High-risk pregnant women and infants: k=3	Yes, GRADE

© 2021 Siddique SM et al. *JAMA Network Open.*

Reference	Objective	Search Timeframe	Inclusion/Exclusion Criteria	Primary Studies: N, Design	Age, No. of studies	Primary dx & comorbidity, No. of studies	GRADE or Similar Analysis
Agarwal 2019			health system utilization		infants: mean range: 23.5 to 28.5		
**Bakker et al. 2011 (32)**	Examine effectiveness of geriatric care teams and units	1980 – May 2009	Included: RCTs and observational studies; patients at least 65 years old; multicomponent interventions Excluded: Non-English studies; single-disease or single-component interventions	17 total studies; 6 reported LOS: 4 RCTs, 2 observational	65+ years	Frail elderly	No
**Eagles et al. 2020 (48)** Note: Lin 2009 also included in Goncalves-Bradley 2016 and Zhu 2015	Examine impact of a geriatric assessment on mortality, hospital length of stay, discharge destination, and delirium incidence in patients 65 years and older admitted to a trauma center	April 26, 2019	Included: Peer-reviewed studies describing impact of geriatric trauma consultation (GTC) in adults 65 years and older admitted to a trauma center compared with standard trauma care alone Excluded: Case reports, SRs, and commentaries	8 retrospective cohort studies (N=122 to 4,534)	Range: 60 to 70 years	Older adults admitted to trauma center	No formal analysis. Authors reported strength of findings are limited by study design, confounding, meta-analysis results based on few studies
**Ellis et al. 2017 (43)** Note: Counsell 2000 also included in Mabire 2018	Examine effectiveness and resource use of comprehensive geriatric assessment (CGA) for older adults admitted to hospital, and	Inception to October 5, 2016	Included: RCTs comparing inpatient CGA versus usual care on a general medical ward or on a ward for older people, usually admitted to hospital for acute care or for inpatient	29 RCTs (N=13,766)	Mean range: 74 to 85 years	Frail or at-risk participants: k=11 Older participants: k=11	Yes, GRADE

© 2021 Siddique SM et al. *JAMA Network Open.*

Reference	Objective	Search Timeframe	Inclusion/Exclusion Criteria	Primary Studies: N, Design	Age, No. of studies	Primary dx & comorbidity, No. of studies	GRADE or Similar Analysis
	to use these data to estimate its cost-effectiveness		rehabilitation after an acute admission				
**Frazer et al. 2019 (44)**	Examine interventions intended to improve safety or quality anticoagulant prescribing	Inception to March 24, 2018	Included: RCTs, non-RCTs, controlled before-after, interrupted time-series in economically developed countries assessing system-level interventions for any indication in adult inpatients aged<br>18 years or older<br>Excluded: Interventions targeting prophylactic (low-dose) anticoagulant use, evaluating intra-operative anticoagulation, delivered in the outpatient setting or at transition to outpatient care, or compared with interventions not in current practice. Cross-sectional, uncontrolled cohort, review articles, unpublished, opinion pieces, conference abstracts/proceedings	19 RCTs (N=12,742)	Mean range: 46 to 77 years	History of AF, ACS, VTE, stroke/TIA, valve replacement, severe heart failure, PVD, bridge to lung transplant, valve disease, systemic arterial embolism, left ventricular thrombus, cardiac prophylaxis	No

© 2021 Siddique SM et al. *JAMA Network Open.*

Reference	Objective	Search Timeframe	Inclusion/Exclusion Criteria	Primary Studies: N, Design	Age, No. of studies	Primary dx & comorbidity, No. of studies	GRADE or Similar Analysis
**Gonçalves-Bradley et al. 2016 (45)**<br><br>*Note: Jack 2009, Kennedy 1987, Laramee 2003, Lin 2009, Naylor 1994, Rich 1995a, Weinberger 1996 also included in Bryant-Lukosius 2015, Zhu 2015, Mabire 2017, Agarwal 2019, and/or Eagles 2020	Examine effectiveness of planning the discharge of individual patients moving from hospital	1946 to October 2015	Included: RCTs; participants were hospital inpatients<br><br>Excluded: Studies of discharge planning part of a broader package of inpatient care, did not describe study design or report control group results	30 RCTs (N=11,964)	75 years: k=10<br><br>70 to<br><br>75 years: k=7<br><br><70 years: k=13	Older participant: k=21<br><br>Mix of medical and surgical conditions including heart failure: k=5<br><br>Psychiatric hospital or general ward: k=2<br><br>Admitted following fall: k=2<br><br>Note: some trials included multiple population types	GRADE (Moderate to very low)
**Gillaizeau et al. 2013 (33)**	Examine effectiveness of computerized advice on drug dosing	Through January 2012	Included: RCTs and observational studies; interventions using computerized advice to guide drug dosing tailored to individual patient<br><br>Excluded: Studies of equations or algorithms not supported by a computerized device; popups or dosing advice that was not patient-specific	42 total studies;<br><br>9 reported LOS:<br><br>8 RCTs,<br><br>1 observational	Not reported	Mix includes diabetes, COPD, renal disease, etc.	Very Low

© 2021 Siddique SM et al. *JAMA Network Open.*

Reference	Objective	Search Timeframe	Inclusion/Exclusion Criteria	Primary Studies: N, Design	Age, No. of studies	Primary dx & comorbidity, No. of studies	GRADE or Similar Analysis
**Huntley et al. 2016 (34)**	Examine effectiveness and cost of case management for patients with heart failure	1985 – November 2015	Included: RCTs and observational studies; adult studies only; all languages	22 total studies; 9 reported LOS: 8 RCTs, 1 observational; 13 reported readmissions: 12 RCTs, 1 observational	65+ years	Congestive heart failure	No
**Kul et al. 2012 (35)**	Examine effectiveness of clinical pathways for patients with heart failure	1985 – 2011	Included: RCTs and observational studies; all languages	7 total studies: 3 RCTs, 1 cohort, 3 pre-post	Mean: >65 years	Congestive heart failure	No
**Mabire et al 2017 (36, 37)** Note: Counsell 2000 also included in Eliis 2017 and Naylor 2004 also included in Bryant-Lukosius 2015	Examine effectiveness of nursing discharge planning interventions on health-related outcomes for older inpatients discharged home	2000 to 2015	Included: Studies published in English of older patients (>65 years) discharged home from an acute care or post-acute care rehabilitation setting, i.e., skilled nursing facility. Interventions had to be provided by at least one nurse and involve a multidisciplinary and/or interdisciplinary model of care	13 total studies: 11 RCTs, 1 pilot cohort, 1 pre-post study (N=3,964)	Median: 77 years	Older patients: With severe comorbidities: k=1 With moderate comorbidities: k=5 With low morbidities: k=5	Yes, GRADE

© 2021 Siddique SM et al. *JAMA Network Open.*

Reference	Objective	Search Timeframe	Inclusion/Exclusion Criteria	Primary Studies: N, Design	Age, No. of studies	Primary dx & comorbidity, No. of studies	GRADE or Similar Analysis
**Patel et al. 2020 (38)**<br>Note: Weingerber 1996 also included in Goncalves-Bradley 2016	Examine treatment of geriatric hip fractures by a multidisciplinary hip fracture service and what impact this has on patient outcomes	January 1, 2012 to November 12, 2017	Included: Indexed in the databases searched, full-text comparative studies published in English that studied at least one of the four main outcome measures of interest	17 total studies:<br>9 retrospective studies,<br>6 prospective studies, 1 RCT,<br>1 non-RCT (N=146 to 23,973)	>60 years	Older patients with hip fracture	Tool used not reported: 16 of<br>17 studies receive an evidence grade of good, 1 of<br>17 received an evidence grade of fair
**Pannick et al. 2015 (39)**	Examine the range of objective patient outcomes used in studies of general medical ward interdisciplinary team care, and to evaluate the performance of interdisciplinary interventions against them	January 1, 1998<br>January 29, 2014	Included: Primary reports of interdisciplinary team care interventions in adult general medical wards using an objective patient outcome measure<br>Excluded: Patients<br><18 years in intensive care unit, operating rooms, stroke units, coronary care, pharmacotherapy; interventions relying solely on a staff member taking dedicated coordinating or facilitating role<br>(e.g., case management); interventions targeting continuation of care by a similar	30 total studies:<br>8 RCTs, 9 cluster-RCTs, 8 non-RCT cluster, 4 before-after, 1 interrupted time series (N=66,548)	Mean:<br>63 years	Variety of primary diagnoses: delirium, community-acquired pneumonia, acute stroke, advanced liver disease, patients taking anticoagulant medication	No

© 2021 Siddique SM et al. *JAMA Network Open.*

Reference	Objective	Search Timeframe	Inclusion/Exclusion Criteria	Primary Studies: N, Design	Age, No. of studies	Primary dx & comorbidity, No. of studies	GRADE or Similar Analysis
			group during the following shift<br>(e.g., handoff processes)				
**Van Craen et al. 2010 (46)**	Examine effectiveness of geriatric evaluation units	Through October 2007	Included: RCTs and cohort studies; patients at least 65 years old; published in English, French, or Dutch<br>Excluded: Studies of single-disease management programs and geriatric consultation services	7 RCTs	65+ years	Frail elderly	No
**White et al. 2011 (40)**	Examine the effectiveness of hospitalists on the quality of inpatient care	1996 – December 2010	Included: All study designs, ages, languages	65 total studies;<br>1 RCT, 8 non-randomized controlled trials,<br>1 interrupted time series, 37 cohort,<br>18 pre-post	All ages	Mix includes heart failure, COPD, psychiatric illness, substance use disorder, etc.	No
**Zhu et al. 2015 (20)**<br>Note: Jack 2009, Lin 2009, and Naylor 1999 also included in Goncalves-Bradley 2016, Eagles 2020, and/or Bryant-	Examine effectiveness of nurse-led early discharge planning program (DPP) to standard care for in-patients with chronic disease or	1946 to<br>March 29, 2014	Included: RCTs; general hospital setting; include at least one primary or secondary outcome<br>Excluded: Non-English studies, assessing patients with acute, critical illness, or social admissions; programs was	10 RCTs (N=3,438)	Mean range:<br>36.4 to<br>94 years	Older hospitalized adults: k=5<br>Decompensated HF: k=1<br>Hip fracture patients: k=1<br>Rehab patients: k=1<br>CHD: k=1	No

© 2021 Siddique SM et al. *JAMA Network Open.*

Reference	Objective	Search Timeframe	Inclusion/Exclusion Criteria	Primary Studies: N, Design	Age, No. of studies	Primary dx & comorbidity, No. of studies	GRADE or Similar Analysis
Lukosius 2015	rehabilitation needs		directed by non-nursing staff; assessing post-discharge care of patients transferred to nursing home or long-term care facility; intervention initiated at discharge; post-discharge care			Hospitalized psychiatric patients: k=1	
**Zhang et al. 2013 (47)**	Examine effectiveness of interventions to prevent postoperative delirium in elderly patients	Through July 2012	Included: RCTs only; adult patients<br>Excluded: Non-English studies; patients with delirium prior to surgery; non-surgical patients; patients with alcohol withdrawal syndrome; studies of homogenous populations of patients with central nervous diseases or mental disorders	38 total RCTs;<br>10 studies reported LOS, only 2 used a systemic intervention (others were pharmacologic)	Mean age: 80+ years	Frail elderly undergoing orthopedic surgery	No

ACS: acute coronary syndrome; AF: atrial fibrillation; AIS: acute ischemic stroke; ARDS: acute respiratory distress syndrome; CGA: comprehensive geriatric assessment; CHD: congenital heart disease; CNS: clinical nurse specialists; COPD: chronic obstructive pulmonary disease; EMR: electronic medical record; GRADE: Grading of Recommendations Assessment, Development and Evaluation; GTC: geriatric trauma consultation; HF: heart failure; LOS: length of stay; PVD: peripheral vascular disease; RCT: randomized controlled trial; TIA: transient ischemic attack; US: United States; VTE: venous thromboembolism

© 2021 Siddique SM et al. *JAMA Network Open.*

eTable 3. Summary of Findings for Length of Stay Meta-analysese

Type of Intervention	Reference	Comparison	Population	Study Design: No. Studies (N)	Findings and Direction of Effect	Strength of Evidence
**Discharge planning**	Mabire et al. 2017 (36, 37)	Nursing discharge planning interventions vs. usual care	Older patients with or without comorbidities	4 RCTs, 1 pre-post, and 1 cohort (3 of 6 US) (n=2,370)	WMD: 0.29 days, 95% CI: 0.24 to 0.35, I^2=0%, intervention increases LOS	Low
**Discharge planning**	Goncalves-Bradley et al. 2016 (45)	Discharge planning vs. usual care	Older patients with a medical condition	12 RCTs (6 of 12 US) (n=2,193)	MD: -0.73 days, 95% CI: -1.33 to -0.12, I^2=9.44%, favors intervention	Moderate
**Discharge planning**	Goncalves-Bradley et al. 2016 (45)	Discharge planning vs. usual care	Older surgical patients	2 RCTs (1 of 2 US) (n=184)	MD: -0.06, 95% CI: -1.23 to 1.11, I^2=0%, no difference	Very Low
**Discharge planning**	Bryant-Lukosius et al. 2015 (42)	Clinical nurse specialists transitional care vs. usual care	Elderly hospitalized patients	3 US RCTs (n=396)	MD: -0.69 days, 95% CI: -1.95 to 0.56, p=0.28, no difference	Low
**Discharge planning**	Bryant-Lukosius et al. 2015 (42)	Clinical nurse specialists transitional care vs. usual care	High-risk pregnant women	2 US RCTs (n=215)	MD: -1.19 days, 95% CI: -1.55 to -0.83, p <0.00001, favors intervention (reduces maternal postpartum LOS)	Moderate
**Discharge planning**	Zhu et al. 2015 (20)	Nurse-led early discharge planning vs. usual care	Mix includes older patients, decompensated heart failure, hip fracture, rehab, congestive heart disease, hospitalized psychiatric patients	5 RCTs (4 of 5 US) (n=1,912)	SMD: 0.03, 95% CI: -0.06 to 0.12, p=0.540, I^2=0%, no difference in LOS	Moderate

© 2021 Siddique SM et al. *JAMA Network Open.*

Type of Intervention	Reference	Comparison	Population	Study Design: No. Studies (N)	Findings and Direction of Effect	Strength of Evidence
**Geriatric assessment**	Eagles et al. 2020 (48)	Geriatric trauma consultation vs. usual trauma care	Older adults admitted to trauma center	2 US retrospective cohort studies (n=5,414)	MD: -1.11 days, 95% CI: -1.43 to -0.79, I^2=0%, favors intervention	Moderate
**Geriatric assessment**	Van Craen et al. 2010 (46)	Geriatric evaluation unit vs. usual care	Frail elderly	7 RCTs (n=4,759)	Mean reduction measured by Hedges g 0.07 days, 95% CI: -0.11 to 0.26, no difference	High*
**Medication management**	Gillaizeau et al. 2013 (33)	Computerized decision support vs. usual care	Mix includes diabetes, COPD, renal disease, etc.	8 RCTs and 1 observational study (n=18,507)	SMD: -0.15, 95% CI: -0.33 to 0.02, I^2=57%, no difference in reduction of LOS, but leans toward favoring intervention	Very Low*
**Clinical pathways**	Kul et al. 2012 (35)	Clinical pathways vs. usual care	Congestive heart failure	1 RCT and 4 observational studies (n=2,095)	Mean reduction: 1.89 days, 95% CI: 1.33 to 2.44, I^2=42%, favors intervention	Low*
**Interdisciplinary care**	Pannick et al. 2015 (39)	Altering interdisciplinary team composition vs. usual care	Mixed patient population – geriatric, liver transplant, psychiatric, delirium, infectious diseases	2 RCTs, 2 non-RCT cluster studies, 2 before/after studies (4 of 6 US) (n=NR)	WMD: 0.087 days, 95% CI: −0.083 to 0.257, no difference	Low*
**Interdisciplinary care**	Pannick et al. 2015 (39)	Altering interdisciplinary team practice vs. usual care	Most studies did not specify patient population, 1 study include geriatric patients and 1	2 cluster RCTs, 3 non RCT cluster studies, 2 interrupted time series (6 of	WMD: 0.001 days, 95% CI: −0.035 to 0.037, no difference	Low*

© 2021 Siddique SM et al. *JAMA Network Open.*

Type of Intervention	Reference	Comparison	Population	Study Design: No. Studies (N)	Findings and Direction of Effect	Strength of Evidence
			study's setting VA hospital	7 US) (n=NR)		
**Case management**	Huntley et al. 2016 (34)	Case management vs. usual care	Congestive heart failure	8 RCTs and 1 observational study (n=1,765)	Mean reduction: 1.28 days, 95% CI: 0.52 to 2.04, I^2=63%, favors intervention Subgroup analysis, excluding studies at high risk of bias: Mean reduction: 1.76 days, 95% CI: 1.23 to 2.29, I^2=14%, favors intervention	Moderate*
**Telehealth**	Baratloo et al. 2018 (31)	Telestroke-based systems vs. bedside (face-to-face)	Tissue plasminogen activator treated patients with acute ischemic stroke	6 retrospective controlled studies, 2 prospective controlled studies, 1 RCT (6 of 9 US) (n=2,850)	MD: -0.55 days, 95% CI: -1.02 to -0.07, p=0.02, I^2=38%, favors intervention	Low*

LOS: length of stay; CI: confidence interval; COPD: chronic obstructive pulmonary disease; MD: mean difference; NR: not reported; OR: odds ratio; RCT: randomized controlled trial; RR: risk ratio; US: United States; VA: Veteran Affairs; WMD: weighted mean difference

*Authors of SRs did not assess the strength of evidence for this outcome. Strength of evidence rating is based on guidance from Berkman et al. 2013 (29).

© 2021 Siddique SM et al. *JAMA Network Open.*
